# Supplementary material for: End Sequence Analysis Toolkit (ESAT) expands the extractable information from single-cell RNA-seq data
Source: Genome Res. 2016 Oct;26(10):1397–410. doi: 10.1101/gr.207902.116 (PMC5052061; doi:10.1101/gr.207902.116)
Supplement: Supplemental Material [file supp_gr.207902.116_Supp_Materials.tar.gz › ESAT-esat_v0.1/doc/html/_transcript_in_genomic_space_8java.html]

ESAT: java/umms/core/coordinatesystem/TranscriptInGenomicSpace.java File Reference


ESAT


- Main Page
- Related Pages
- Packages
- Classes
- Files

- File List

All Classes Namespaces Files Functions Variables Enumerator Pages

- java
- umms
- core
- coordinatesystem

Classes |
Packages

TranscriptInGenomicSpace.java File Reference

|  |  |
| --- | --- |
| Classes | |
| class | umms.core.coordinatesystem.TranscriptInGenomicSpace |
|  | |

|  |  |
| --- | --- |
| Packages | |
| package | umms.core.coordinatesystem |
|  | |


---

Generated on Fri Aug 15 2014 15:10:49 for ESAT by  

 1.8.7
